# Group conquers efficacy: Preschoolers’ imitation under conflict between minimal group membership and behavior efficacy

**DOI:** 10.1371/journal.pone.0223101

**Published:** 2019-09-26

**Authors:** Yuanyuan Li, Yifan Liao, Yuang Cheng, Jie He

**Affiliations:** Department of Psychology and Behavioral Sciences, Zhejiang University, Hangzhou, China; University of Queensland, AUSTRALIA

## Abstract

Research has found that preschoolers’ imitation demonstrates in-group bias and is guided by behavior efficacy. However, little is known about whether children’s sensitivity to behavior efficacy affects their in-group imitation. This study aimed to investigate preschoolers’ imitation tendency when group preference and behavior efficacy are in conflict. Participants were 4-year-old (*N* = 72) and 6-year-old (*N* = 72) preschoolers in China. They observed two demonstrators (one in-group and one out-group) pressing two different buttons, respectively, to turn on a music box, and were then asked to try it themselves. In the experimental condition, the out-group demonstrator always succeeded, whereas the in-group demonstrator failed half the time. The results showed that more 6-year-old children imitated the less-effective behaviors of the in-group demonstrator, whereas 4-year-old children showed no such inclination. Two control conditions confirmed that children chose to imitate in-group rather than out-group members (Control 1: both in-group and out-group demonstrators succeeded all four times), and could imitate according to efficacy (Control 2: two in-group demonstrators succeeded two and four times, respectively). These results indicated that 6-year-olds faithfully followed the in-group modeled behavior, regardless of behavior efficacy. Results are discussed through the social function of in-group imitative learning.

## Introduction

As social animals, human beings have evolved to live in complex societies and spend a great deal of time interacting with others [1]. Group becomes an important medium for people to maintain connection with others and gain a sense of belonging [2]. Children acquire the concept of “us” and “them” at an early age. They show social preferences for in-groups of the same age, gender [3], race [4], and language [5]. However, it is difficult to determine whether preferences for these in-groups are caused by familiarity, cultural stereotypes, or in-group bias alone. To find the baseline conditions of in-group bias, Tajfel and colleagues designed the “minimal group” paradigm, in which teenagers were arbitrarily assigned to different groups, either at random or according to esthetic preferences, and found that arbitrary minimal groupings were sufficient to induce in-group bias [6, 7]. The robust effect of minimal in-group bias was verified across various measures referring to a meta-analytic review [8]. Evolutionary evidence indicates that in-group preference evolves to be normative because it promotes within-group cooperation that can bring practical benefits to each group member and guarantee group continuation [9, 10]. Developmental research also found minimal in-group bias in young children [11–14]. In the work of Dunham and colleagues [14], children were asked to explicitly rate their liking of a target and to participate in an Implicit Association Test (IAT) to indicate their implicit attitudes. Results found that children preferred in-group members both implicitly and explicitly, and that implicit minimal in-group preference was stronger than explicit in-group preference [14].

Notably, group membership also guides social learning behavior in imitation tasks [11, 12]. For example, Wilks and colleagues [11] reported that children imitated even antisocial in-group members. Children were exposed to in-group and out-group members engaging in prosocial (sharing or helping) and antisocial (not sharing or hindering) behaviors, and then participated in an imitation task. Results found that children persisted in imitating in-group members, even when they had behaved antisocially [11]. Furthermore, children imitate in-group members especially when they are excluded by the in-group. In the work of Watson-Jones and colleagues [12], children were included or excluded by in-group or out-group members while playing the Cyberball game. Results demonstrated that children who were excluded by in-group members more faithfully copied their in-group models’ meaningless actions. This high-fidelity imitation was discussed as an approach to gain reinclusion [12].

According to the evidence above, children demonstrate intense in-group bias in imitation. Imitation is defined as involving the recognition and reproduction of a goal as well as the specific actions that brought about the goal [15]. Because the efficacy of various actions toward the same goal differ, if the desired outcome of an action is rarely repeated, children should recognize the poor efficacy of the action and reduce their imitation fidelity [16]. Evidence indicates that children grasp the concept of probability [17–19], the Sampling Hypothesis [20], and Bayesian inference [21, 22], all of which are important tools for understanding efficacy. Although many studies have demonstrated that children can flexibly adjust their learning behaviors according to a model’s professionalism and credibility [23–25] and the children’s own experience of task difficulty [26, 27], few studies have focused on how the observed efficacy of an action affects children’s selective imitation [16]. That is, if the observed action does not always produce the goal, will children alter their imitative fidelity? One study suggested that both toddlers and preschoolers could utilize efficacy information to guide their imitation. Children were shown a demonstrator who activated a music box either in every instance or in some instances. Results found that children imitated the deterministically effective actions more faithfully than the probabilistically effective actions [28].

In general, children tend to imitate in-group models and more efficient models. The present study aimed to investigate whether children’s sensitivity to behavior efficacy would affect their in-group bias in imitation. This issue is important because, in everyday life and in the course of history, the out-group often adopts more effective methods or more advanced tools than the in-group. For example, Hoffman mentioned that in the 16^th^ century, the French and English armies adopted the Spanish infantry’s discipline, training, and small team cohesion when they found out-group (Spanish) organization was more effective, and that military technology had improved via such “learning by doing” [29]. To examine how children trade off group membership and behavior efficacy, the present study set up an experimental condition in which the out-group method was more effective than the in-group method, and investigated whether children would copy the more advanced “others” or insist on following the less-effective in-group model.

Some studies address issues similar to the trade-off between group membership and behavior efficacy. For example, in studies of “overimitation,” although children knew that some of the demonstrator’s actions had no effect on the outcomes, they still copied all of the demonstrator’s unnecessary actions [30]. Gruber and colleagues found that children overimitated more often when unnecessary actions were performed by an in-group rather than an out-group model [31]. However, “unnecessary actions” describes low behavior efficiency, which means the actions have no causal relevance to the goal; in such cases, the goal has already been achieved [32]. Conversely, low behavior efficacy means the incompletion of the goal; that is, the goal has not been achieved. The interest of the present research was to investigate whether children’s attitudes toward efficacy influence their in-group imitation. Other studies discuss children’s imitative trade-off between majority and efficacy. For instance, preschoolers showed an inclination to copy the majority when both the majority and individuals were successful, but preferred to copy successful individuals when the majority were unsuccessful [33]. That is, high-efficacy bias could trump majority bias. However, majority bias itself is a heuristic that helps individuals assess behavior value based on frequency, and it offers some amount of probability information. When the modeled behavior did not work in three consecutive instances, it was seen as more likely to not work at all, and children refused to imitate it. In contrast, the present research manipulated the less-efficient models so that they did succeed sometimes.

The question of when children begin to show minimal group bias remains controversial. Most research has found minimal group bias only in children older than 5 [2, 14, 34]. However, Richter and colleagues provided multiple “minimal” grouping cues (an armband, a sticker, and a scarf) and found that even 3-year-olds preferred in-group members [13]. Furthermore, regarding possible age differences, evidence indicates that older children seemed to imitate in-group models regardless of the context compared to younger children [35]. To verify the results of previous research and to explore age differences, two age groups (4- and 6-year-olds) were recruited to participate in the present study. Children were asked to operate the music box after observing two demonstrators repeatedly attempting to activate it. In the experimental condition, in-group models were designed to succeed probabilistically, and out-group models were designed to succeed deterministically. We anticipated that children, especially older children, would imitate the in-group model regardless of efficacy.

## Materials and methods

### Participants

Seventy-two 4-year-olds (mean age = 4.40 years; range = 3.72–4.76; 32 girls) and seventy-two 6-year-olds (mean age = 5.69 years; range = 5.13–6.20; 37 girls) were recruited from two kindergartens in Hangzhou, a large city located in the southeast of China. All parents had a high school level of education or higher. All children were of Han ethnicity. Written consent was obtained from the participants’ parents. The study was approved by the Institutional Review Board of the Department of Psychology and Behavioral Sciences of Zhejiang University. Both age groups were randomly assigned to one of three conditions (one experimental condition and two control conditions), with 24 children allocated to each. The sample size was verified using G*Power 3. Referring to previous research [14], the ratio of participants who preferred the in-group to that of participants who preferred the out-group was 2.8, yielding a sample size of 23. Six additional children were tested but excluded because of experimenter error (1), attention deficit (1), memory error (2), or failure to be grouped (2).

### Materials

**Fig 1:**
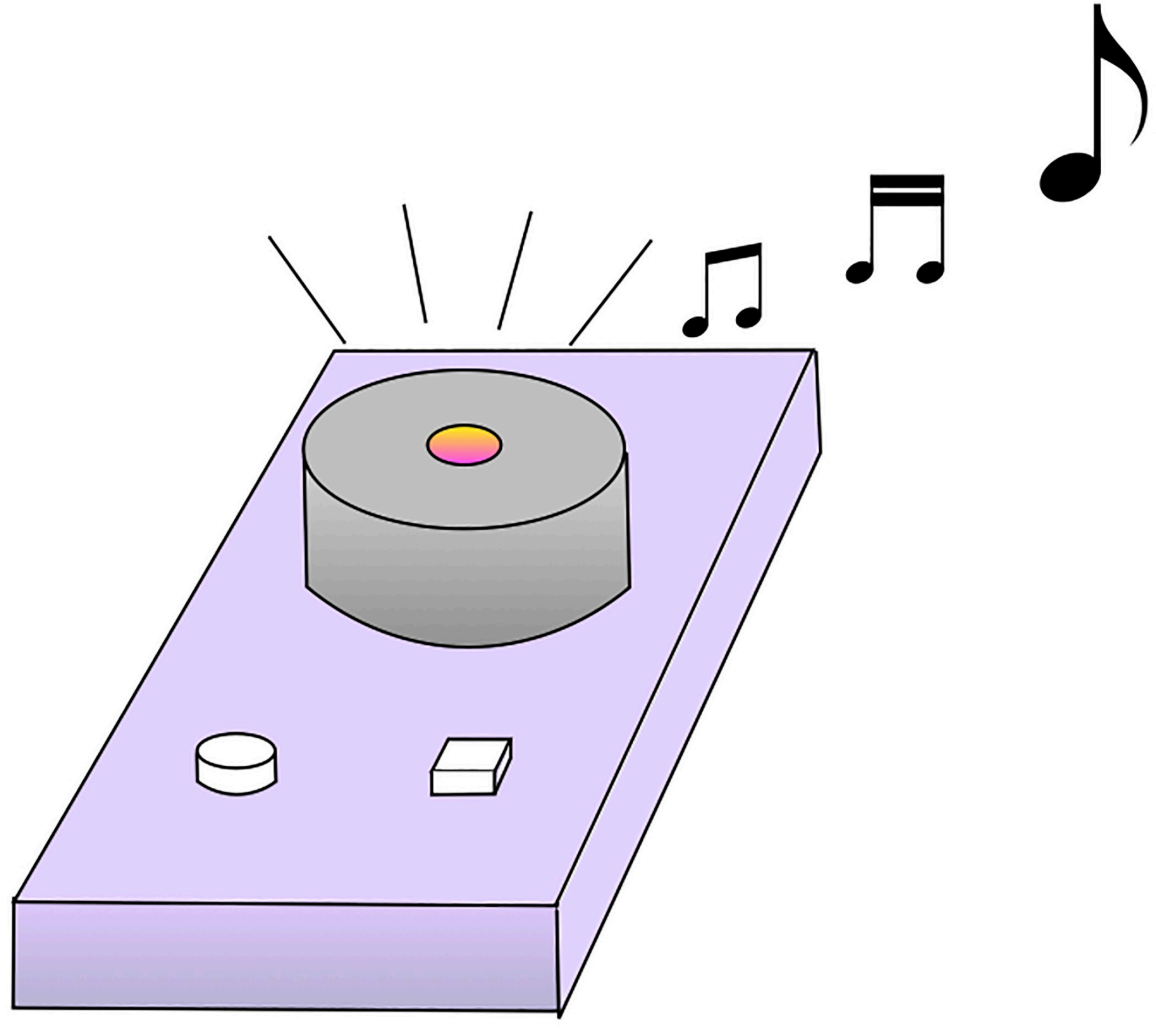
Sketch of the remote-controlled music box used in the experiment.

A remote-controlled music box similar to the one used in Schulz and colleagues’ research was used to manipulate success probability [28]. The box was 20 cm × 14 cm × 7 cm (Fig 1) with two buttons, one round and the other square. Participants were told that the music box was controlled by the buttons; however, it was actually activated by a remote control. When activated, the music box would flash and play music. Blue or yellow T-shirts, caps, and wristbands were used to divide the children into either a blue or a yellow group, and a laptop was used to play a demonstration video.

### Procedure

All participants were randomly assigned to one of the three conditions (one experimental condition and two control conditions) and tested individually in a quiet room. In the *experimental condition*, the out-group demonstrator’s success rate was higher than the in-group demonstrator’s. In the *group control condition*, both in-group and out-group demonstrators succeeded all the time, to ensure that the children indeed imitated in-group modeled behaviors. In the *efficacy control condition*, both demonstrators were from the in-group but had different success rates, to ensure that the children imitated the more effective behaviors. All other components were the same across the three conditions, except for the video demonstration component.

#### Grouping

In the experimental and group control conditions, children were assigned to the yellow or the blue group. Grouping was randomly pre-arranged on the recording paper before the experiment. Both an in-group and an out-group demonstrator were present. The in-group demonstrator wore the same color as the children, and the out-group demonstrator wore the opposite color. In the efficacy control condition, both demonstrators were in-group members. Children were asked to wear caps and wristbands of the corresponding color to ensure in-group identity.

#### Group-preference training task

The training process in Watson-Jones and colleagues’ research was used to ensure group identity [12]. After being assigned to the yellow or the blue group, children participated in the training task to ensure that they exhibited preferences similar to their in-group members. Participants were shown a picture with two characters, a boy and a girl, wearing clothing and holding balloons of the same color as the participants. They were told that these two characters were their in-group members. The next picture showed a dog, a cat, and a horse. Children were asked, “Which is your favorite animal?” After choosing one animal, children were shown that the two in-group characters also liked the same animal they did. Two additional analogous processes with fruits and toys were employed to help the children confirm their similarities with their in-group members.

#### Video demonstration

To ensure that the children knew to which group they belonged, the experimenter played a demonstration video. To exclude the influence of gender bias, girls and boys watched a girls’ and boys’ demonstration video, respectively. Each child saw two demonstrations (in the experimental and group control conditions, one from the in-group and one from the out-group demonstrator; in the efficacy control condition, both from in-group demonstrators) in a counterbalanced order. The videos began with the demonstrators saying, “Hello,” then telling the children to carefully watch how they could turn on the music box. They pressed one button and said, “One!” as the music box simultaneously was activated (or not). After three seconds, the music stopped. The demonstrators would repeat the same process, pressing the same button, for another three instances, and counting respectively, “Two!”, “Three!”, “Four!”. The two demonstrators would press different buttons, and the music box would or would not activate, according to the following conditions. In the experimental condition, the out-group demonstrator always succeeded, whereas the in-group demonstrator failed two out of four times. In the group control condition, both the in-group and out-group demonstrators succeeded all four times. In the efficacy control condition, one in-group demonstrator succeeded all the time, whereas the other in-group demonstrator succeeded two out of four times.

#### Imitation task

As soon as the demonstration video ended, the music box was placed on the table and the children were told, “Now it is your turn, and you have only one chance. Please try it!” Only the children’s first choice was recorded, and the music box was activated as long as they pressed either of the buttons. Notably, we only recorded which button the child pressed without coding their specific hand movements. It may be controversial to call such a task “imitation” as the notion refers to copying the form of an action [36]. Here, we adopted a more generalized application of “imitation,” which refers to following the model’s behavioral choice.

After the children gave their responses, they were asked why they chose that button. Explanations were coded into three categories according to the information children mentioned: (a) group (i.e., children chose the button for the sake of their in-group preference or keeping in-group consistency; e.g., “I am in the yellow group,” “Yellow group members should press this button.”), (b) efficacy (i.e., children chose the button because they found it more effective or they cared about the result; e.g., “It is more convenient,” “It turns on every time this button is pressed.”), or (c) absent or irrelevant response (e.g., “I don’t know,” “I like this button,” “She pressed this button just now.”) All answers were recorded and independently coded later by two naïve coders. Coders reached a high degree of agreement (Cohen’s κ = 0.895). Disagreements were resolved through discussion. Finally, to test the transferring effect of minimal in-group bias and proficiency bias, children were asked, “Which demonstrator do you prefer?” and “Which one do you think is cleverer?” The memory check question “Which button did they press respectively just now?” was asked to ensure that the children remembered the modeled behaviors.

#### Data analysis

In this section, we detail the process of data analysis. First, to examine whether 4- or 6-year-old children in each condition chose the prospective demonstrator more often, we compared the number of children who chose the in-group (or the more effective) demonstrator with the chance level of 12 (50%) using the Chi-square test. Second, we used generalized linear mixed models (GLMMs, via SPSS version 23) with a binomial distribution to examine whether age, group membership, or behavior efficacy affected children’s choices. Previous research has reported a gender effect in overimitation tasks; therefore, gender was also included in the model [37]. Models were performed on each dependent variable (children’s choices in imitation, preference, or cleverness attribution tasks), with age (4 vs. 6), gender, condition (experimental vs. efficacy control, and experimental vs. group control), and their interaction as fixed factors, with participants’ identity as a random factor. All interactions were included in the initial model and insignificant interactions were removed from the final model subsequently. Only the final model will be reported in results. Because the manipulated variables in two control conditions were very different, two separate models were run to compare children’s choices in the efficacy or group control condition with choices in the experimental condition on each dependent variable (imitation, preference, or trait attribution).

## Results

### Imitation

**Fig 2:**
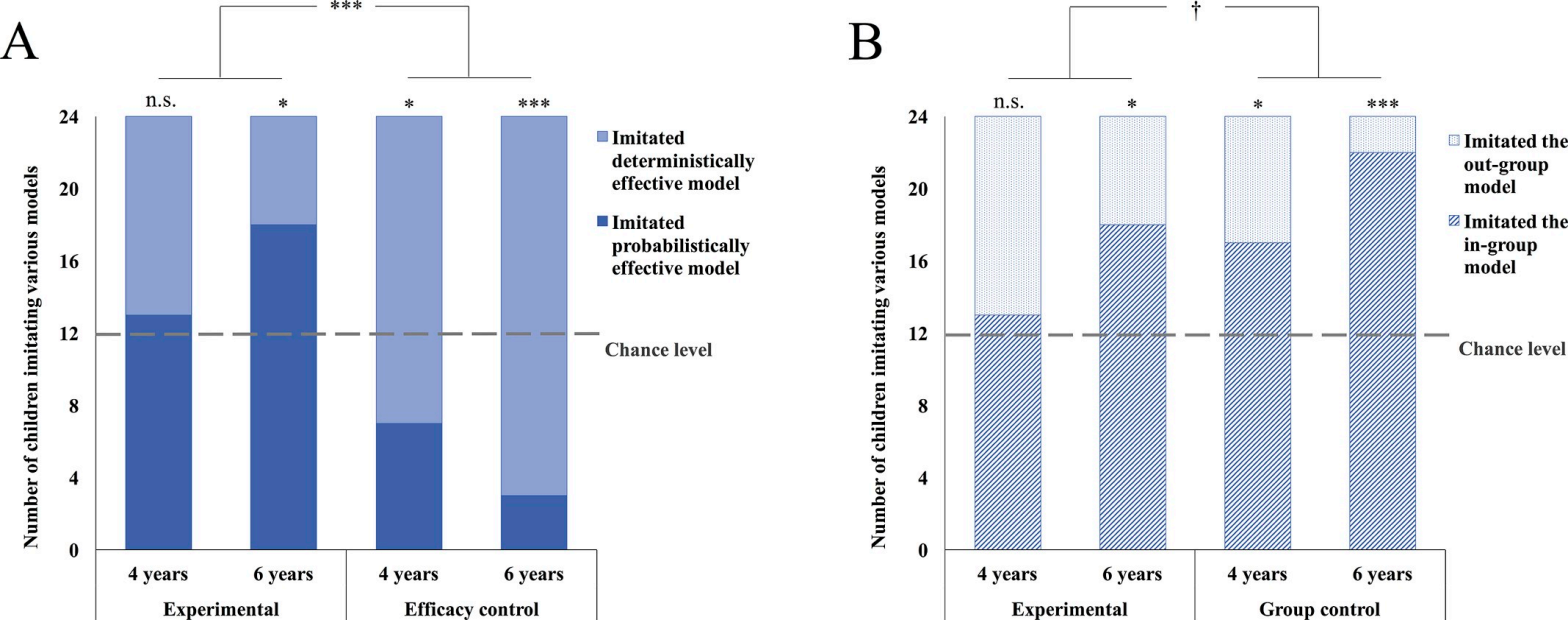
Number of children who imitated the different model behaviors. (A) Number of children who imitated the deterministically and probabilistically effective models in the experimental and efficacy control conditions. (B) Number of children who imitated the out-group and in-group models in the experimental and group control conditions. ^†^*p* < .1, *p < .05, ***p < .001.

In the experimental condition (Fig 2), 4-year-old children showed no inclination between the in-group and out-group demonstrators (54.2%, χ^2^ = 0.167, *p =* .683) whereas 6-year-olds significantly inclined to imitate in-group demonstrators even though they succeeded less frequently than out-group demonstrators (75.0%, χ^2^ = 6.000, *p* = .014). As expected, in the efficacy control condition (Fig 2A), during which two in-group models pressed different buttons with high or low success rates, both age groups chose to imitate the demonstrator who always succeeded (4-year-olds 70.8%, χ^2^ = 4.167, *p* = .041; 6-year-olds 87.5%, χ^2^ = 13.500, *p* = .000). In the group control condition (Fig 2B), in which both in-group and out-group models succeeded deterministically, both 4-year-old and 6-year-old children preferred to imitate in-group demonstrators (4-year-olds 70.8%, χ^2^ = 4.167, *p* = .041; 6-year-olds 91.7%, χ^2^ = 16.667, *p* = .000). That is, children as young as 4 years old could understand the experiment setup and use both group and efficacy information to guide their imitation.

The GLMM including children’s imitative performance in the experimental condition and the efficacy control condition showed a significant effect of condition, *F* (1, 91) = 16.580, *p* = .000; and a weak trend of interaction between age and condition, *F* (1, 91) = 3.494, *p* = .065. These results indicated that when the more effective demonstrator belonged to the out-group in the experimental condition, children imitated their behaviors less often than in the efficacy control condition where both demonstrators were in-group members. Moreover, the effect tended to be stronger among 6-year-olds. Neither the effect of age, *F* (1, 91) = 0.023, *p* = .881, nor the effect of gender, *F* (1, 91) = 0.783, *p* = .379, was significant. Another GLMM including data in the experimental condition and the group control condition showed that older children imitated the in-group demonstrator more often, *F* (1, 92) = 4.059, *p* = .047. Furthermore, there was a weak trend that more children imitated the in-group demonstrator in the group control condition than in the experimental condition, *F* (1, 92) = 3.765, *p* = .055. There was no gender effect, *F* (1, 92) = 3.040, *p* = .085.

After imitating the behaviors, children were asked why they made their choices (Table 1). Results showed that in the experimental condition, both age groups showed no difference in referring to group or efficacy (4-year-olds χ^2^ = 2.000, *p* = .157; 6-year-olds χ^2^ = 2.882, *p* = .090). In the group control condition, 6-year-olds focused more on group information (χ^2^ = 14.222, *p* = .000), whereas 4-year-olds only showed a weak trend towards group information (χ^2^ = 3.769, *p* = .052). And in the efficacy control condition, both age groups focused more on efficacy information (4-year-olds χ^2^ = 10.000, *p* = .002; 6-year-olds χ^2^ = 14.000, *p* = .000).

**Table 1:** Children’s reasons for their imitation choice (three categories).

	Age	Group	Efficacy	Absent or irrelevant response
**Experimental**	4	12 (50.0)	6 (25.0)	6 (25.0)
	6	12 (50.0)	5 (20.8)	7 (29.2)
**Group control**	4	10 (41.7)	3 (12.5)	11 (45.8)
	6	17 (70.8)	1 (4.2)	6 (25.0)
**Efficacy control**	4	0 (0)	10 (41.7)	14 (58.3)
	6	0 (0)	14 (58.3)	10 (41.7)

Percentages are in parentheses.

### Preference

Children were also asked their preferences for the two demonstrators. In the experimental condition, when in-group models showed low behavior efficacy, 4-year-olds did not show any significant preference (65.2% of 23 children preferred the in-group demonstrator, χ^2^ = 2.130, *p* = .144), whereas 6-year-olds preferred in-group models over out-group models (86.4% of 22 children, χ^2^ = 11.636, *p* = .000). In the group control condition, both age groups showed preferences for in-group demonstrators (4-year-olds 78.3% of 23 children, χ^2^ = 7.348, *p* = .007; 6-year-olds 91.3% of 23 children, χ^2^ = 15.696, *p* = .000). Whereas in the efficacy control condition, only 6-year-olds showed preferences for demonstrators who deterministically activated the music box (91.3% of 23 children, χ^2^ = 15.696, *p* = .000). Four-year-olds showed no significant inclination in their preferences (66.7% of 24 children preferred those who always succeeded, χ^2^ = 2.667, *p* = .102).

The GLMM with children’s preference choices in the experimental and the efficacy control conditions yielded a significant effect of condition, *F* (1, 87) = 22.300, *p* = .000; and an interaction between age and condition, *F* (1, 87) = 6.338, *p* = .014. The results indicated that more children in the efficacy control condition preferred the effective demonstrator than those in the experimental condition where the effective demonstrator belonged to the out-group, and this effect was stronger among 6-year-olds. The effect of age, *F* (1, 87) = 0.111, *p* = .739; and gender, *F* (1, 87) = 0.487, *p* = .487, was not significant. The GLMM including data in the experimental and the group control conditions showed that more 6-year-olds preferred the in-group demonstrator than 4-year-olds, *F* (1, 86) = 4.173, *p* = .044. Neither the effect of gender, *F* (1, 86) = 0.098, *p* = .755, nor the effect of condition, *F* (1, 86) = 1.608, *p* = .208, was found. However, there was an interaction between gender and condition, *F* (1, 86) = 4.041, *p* = .048. Namely, compared to group control condition in which both the in-group and the out-group demonstrators succeeded all the time, more girls no longer preferred the in-group demonstrators when they succeeded less often in the experimental condition.

### Cleverness attribution

Children were also asked “Which one do you think is cleverer?” In the experimental condition, both age groups thought that the in-group models were as clever as the out-group models (4-year-olds 40.9% of 22 children, χ^2^ = 0.727, *p* = .394; 6-year-olds 40.0% of 20 children, χ^2^ = 0.800, *p* = .371). In the group control condition, both age groups thought the in-group demonstrators cleverer than the out-group demonstrators (4-year-olds 72.7% of 22 children, χ^2^ = 4.545, *p* = .033; 6-year-olds 76.2% of 21 children, χ^2^ = 5.761, *p* = .016). And in the efficacy control condition, both age groups showed no inclination in attribution of cleverness (4-year-olds 66.7% of 24 children, χ^2^ = 2.667, *p* = .102; 6-year-olds 65.2% of 23 children thought the deterministically successful models cleverer, χ^2^ = 2.130, *p* = .144).

The same GLMM containing cleverness attribution data in the experimental and the efficacy control conditions were performed, and no significant effect was found with age, *F* (1, 85) = 0.001, *p* = .981; gender, *F* (1, 85) = 0.020, *p* = .887; and condition, *F* (1, 85) = 0.378, *p* = .540. Another GLMM containing data in the experimental and the group control conditions indicated that when the in-group demonstrators failed several times in the experimental condition, fewer children thought that they were the cleverer among the two demonstrators compared to the group control condition, *F* (1, 81) = 9.079, *p* = .003. Neither the effect of age, *F* (1, 81) = 0.025, *p* = .875, nor the effect of gender, *F* (1, 81) = 0.037, *p* = .848, was significant.

## Discussion

The present study investigated how children imitated behavior when they observed contradictions between group membership and behavior efficacy. When children were exposed to demonstrations in which the in-group model succeeded less than the out-group model, 4-year-olds showed no inclination in imitation, whereas significantly more 6-year-olds imitated the in-group model. The present study extends previous results on in-group bias in imitation by showing that children persisted on following the in-group model, even at the expense of behavior efficacy.

Above all, both older children (6-year-olds) and younger children (4-year-olds) in the present study showed in-group bias, consistent with the results of Richter and colleagues’ study [13]. The inefficacy of the in-group demonstrators had little effect on children’s in-group bias in imitation. Although children were able to imitate the deterministically effective demonstrators in the efficacy control condition, when the deterministically effective demonstrators belonged to the out-group in experimental condition, fewer children chose to imitate them. Wilks and colleagues [11] reported similar in-group bias behaviors in imitation. In their study, although children liked in-group members who behaved antisocially at reduced rates, they insisted on imitating them behaviorally. One possible explanation suggested that children’s reported preference and imitative inclination were motivated by different drives. Liking was guided by kindness, and imitation by perception of competence [11]. However, this hypothesis cannot be directly verified, as both methods modeled in the study were equally effective. In contrast, the present study manipulated behavior efficacy to show low competence in the in-group demonstrator. Nevertheless, children still chose to imitate the in-group model and appeared to be faithful in-group followers, regardless of behavior efficacy.

The phenomenon presented here is of great practical significance. For children themselves, faithfulness to an in-group method could impede their opportunity to learn new things, but can help connect them to the group. From the broader perspective of the group, new technology may meet more cautious acceptance, whereas convention and culture are inherited easily [38]. In that way, the following question is thought-provoking, why do children show such striking in-group bias in imitation? Several explanations are possible. First, because imitation can increase rapport between the demonstrator and the imitator [39], it is possible that children imitate the in-group member to gain their group’s acceptance and favor [12, 38]. Additionally, even if the in-group modeled behavior turns out to be wrong, diffusion of responsibility means that the group follower does not have to take responsibility for the failure.

More importantly, the current results yielded interactions between age and condition (significant in preference and marginal significant in imitation), indicating that out-group membership reduced children’s preference for the more efficient demonstrator, and the effect was stronger among 6-year-olds. Similarly, the result regarding age also showed that more 6-year-olds chose to imitate the ineffective in-group modeled behaviors, whereas 4-year-olds showed no inclination in the experimental condition. This result is not likely to be due to 6-year-olds’ lack of awareness or ignorance of efficacy information. First, both age groups were inclined to imitate the deterministically effective demonstrators in the efficacy control condition, demonstrating the children’s understanding of efficacy manipulation. Second, compared to the group control condition, when the in-group behavior failed two of four times in the experimental condition, neither age group thought that the in-group demonstrator was any cleverer. These findings suggest that both age groups noticed the efficacy information in experimental condition. However, the two age groups weighed efficacy and group membership differently. Older children showed stronger in-group bias and considered the in-group’s behaviors to be normative, but it was not the case for younger children. In previous studies, although there was no preference training after grouping, young children still chose to follow the in-group model [14, 31]. In the present research, however, even preference training was not enough to impel 4-year-olds to imitate (or prefer) the in-group model who was inefficient. The results implied that 4-year-olds valued both group membership and behavior efficacy and vacillated between them.

Interestingly, research concerning other social learning forms reported similar age differences, which indirectly confirmed our finding. For example, in a strong conformity task, 5-year-olds, not younger children, construed conformity as a strategy to win group acceptance [40]. Moreover, considering the research on overimitation, older children imitated more unnecessary behaviors than young children [41, 42]. Ontogenetic evidence also demonstrated that in-group favoritism preceded out-group derogation in the order of development, and when out-group derogation had developed, children would show stronger in-group bias [43].

Previous research has confirmed children’s minimal in-group preference in numerous tasks such as implicit attitude, behavior attribution [14], helping [2], and learning behaviors [11, 12]. The present study found similar effects, in that not only did children imitate the in-group members behaviorally, they also preferred them emotionally and thought them clever cognitively. However, when the in-group model succeeded less often in the experimental condition, the number of children who thought them cleverer declined but was still around chance level. In other words, children refused to admit that the out-group was more intelligent than they were based on efficacy. Likewise, in the efficacy control condition, efficacy also had no effect on cleverness attribution. High-efficacy bias appeared only in children’s imitative behaviors and older children’s preferences. More evidence is needed to verify these results, especially whether high-efficacy bias appears in tasks other than imitation or learning. Another interesting question is whether children will imitate the in-group if the in-group efficacy is even lower. In the present work, we only set conditions of 50% and 100%. However, 50% was still too high for children to refuse since not turning on a music box brought no harm to anybody. Subsequent studies should consider lower success rates such as 25%.

It is important to explore what else, beyond efficacy, children view as worth sacrificing to align with the group. For example, teenagers take up smoking and young children scorn someone just because their group does. They are willing to align with their group members even when they know it will cause harm to themselves or others. Further research should focus on when and where similar sacrifices are made, which will provide a better understanding of the universality and complexity of in-group bias.

Overall, group membership plays an important role in children’s imitation; even behavior efficacy can be sacrificed to follow the in-group model. Even when group membership was artificially assigned in a laboratory setting, even when the in-group demonstrator failed half of the time, 6-year-old children still copied the in-group model’s behavior to gain group affiliation whereas 4-year-olds did not. Children’s faithful in-group imitation may be a key element of human survival in their large and complex society. The present study provides evidence that group membership overrides behavior efficacy in children’s imitative learning.

## References

[1] Aronson E. The social animal. 11th ed New York: Worth; 2011.

[2] Plötner M, Over H, Carpenter M, Tomasello M. The effects of collaboration and minimal-group membership on children’s prosocial behavior, liking, affiliation, and trust. Journal of Experimental Child Psychology. 2015;139:161–73. 10.1016/j.jecp.2015.05.008 26112747

[3] Shutts K, Banaji MR, Spelke ES. Social categories guide young children's preferences for novel objects. Developmental Science. 2010;13(4):599–610. 10.1111/j.1467-7687.2009.00913.x 20590724 PMC2898520

[4] Kelly DJ, Quinn PC, Slater AM, Kang L, Ge L, Pascalis O. The Other-Race Effect Develops during Infancy: Evidence of Perceptual Narrowing. Psychological Science. 2007;18(12):1084–9. 10.1111/j.1467-9280.2007.02029.x 18031416 PMC2566514

[5] Kinzler KD, Dupoux E, Spelke ES. The native language of social cognition. Proceedings of the National Academy of Sciences of the United States of America. 2007;104(30):12577–80. 10.1073/pnas.0705345104 17640881 PMC1941511

[6] Tajfel H, Billig MG, Bundy RP, Flament C. Social categorization and intergroup behaviour. European Journal of Social Psychology. 1971;1(2):149–78. 10.1002/ejsp.2420010202

[7] Billig M, Tajfel H. Social categorization and similarity in intergroup behaviour. European Journal of Social Psychology. 1973;3(1):27–52. 10.1002/ejsp.2420030103

[8] Mullen B, Brown R, Smith C. Ingroup bias as a function of salience, relevance, and status: An integration. European Journal of Social Psychology. 1992;22(2):103–22. 10.1002/ejsp.2420220202

[9] McAuliffe K, Dunham Y. Group bias in cooperative norm enforcement. Philosophical Transactions of the Royal Society B: Biological Sciences. 2016;371(1686):20150073 10.1098/rstb.2015.0073 26644592 PMC4685519

[10] Bowles S. Group Competition, Reproductive Leveling, and the Evolution of Human Altruism. Science. 2006;314(5805):1569–72. 10.1126/science.1134829 17158320

[11] Wilks M, Kirby J, Nielsen M. Children imitate antisocial in-group members. Developmental Science. 2018;21(6):e12675 10.1111/desc.12675 29691975

[12] Watson-Jones RE, Whitehouse H, Legare CH. In-Group Ostracism Increases High-Fidelity Imitation in Early Childhood. Psychological Science. 2016;27(1):34–42. 10.1177/0956797615607205 26573906

[13] Richter N, Over H, Dunham Y. The Effects of Minimal Group Membership on Young Preschoolers’ Social Preferences, Estimates of Similarity, and Behavioral Attribution. Collabra. 2016;2(1):1–8. 10.1525/collabra.44

[14] Dunham Y, Baron AS, Carey S. Consequences of "Minimal" Group Affiliations in Children. Child Development. 2011;82(3):793–811. 10.1111/j.1467-8624.2011.01577.x 21413937 PMC3513287

[15] Want SC, Harris PL. How do children ape? Applying concepts from the study of non-human primates to the developmental study of ‘imitation’ in children. Developmental Science. 2002;5(1):1–14. 10.1111/1467-7687.00194

[16] Carr K, Kendal RL, Flynn EG. Imitate or innovate? Children’s innovation is influenced by the efficacy of observed behaviour. Cognition. 2015;142:322–32. 10.1016/j.cognition.2015.05.005 26072278

[17] Kushnir T, Gopnik A. Conditional probability versus spatial contiguity in causal learning: Preschoolers use new contingency evidence to overcome prior spatial assumptions. Developmental Psychology. 2007;43(1):186–96. 10.1037/0012-1649.43.1.186 17201518

[18] Kushnir T, Gopnik A. Young children infer causal strength from probabilities and interventions. Psychological Science. 2005;16(9):678–83. 10.1111/j.1467-9280.2005.01595.x 16137252

[19] Bonawitz EB, Lombrozo T. Occam's rattle: Children's use of simplicity and probability to constrain inference. Developmental Psychology. 2012;48(4):1156–64. 10.1037/a0026471 22201450

[20] Denison S, Bonawitz E, Gopnik A, Griffiths TL. Rational variability in children’s causal inferences: The Sampling Hypothesis. Cognition. 2013;126(2):285–300. 10.1016/j.cognition.2012.10.010 23200511

[21] Schulz LE, Bonawitz EB, Griffiths TL. Can being scared cause tummy aches? Naive theories, ambiguous evidence, and preschoolers' causal inferences. Developmental Psychology. 2007;43(5):1124–39. 10.1037/0012-1649.43.5.1124 17723040

[22] Gopnik A, Glymour C, Sobel DM. A theory of causal learning in children: causal maps and Bayes nets. Psychological Review. 2004;111(1):3 10.1037/0033-295X.111.1.3 14756583

[23] Koenig MA, Clément F, Harris PL. Trust in Testimony: Children's Use of True and False Statements. Psychological Science. 2004;15(10):694–8. 10.1111/j.0956-7976.2004.00742.x 15447641

[24] Birch SAJ, Vauthier SA, Bloom P. Three- and four-year-olds spontaneously use others’ past performance to guide their learning. Cognition. 2008;107(3):1018–34. 10.1016/j.cognition.2007.12.008 18295193

[25] VanderBorght M, Jaswal VK. Who knows best? Preschoolers sometimes prefer child informants over adult informants. Infant & Child Development. 2009;18(1):61–71. 10.1002/icd.591 20047013 PMC2677762

[26] Williamson RA, Meltzoff AN, Markman EM. Prior experiences and perceived efficacy influence 3-year-olds' imitation. Developmental psychology. 2008;44(1):275–85. 10.1037/0012-1649.44.1.275 18194026 PMC2259446

[27] Williamson RA, Meltzoff AN. Own and others’ prior experiences influence children's imitation of causal acts. Cognitive Development. 2011;26(3):260–8. 10.1016/j.cogdev.2011.04.002 21966091 PMC3181112

[28] Schulz LE, Hooppell C, Jenkins AC. Judicious Imitation: Children Differentially Imitate Deterministically and Probabilistically Effective Actions. Child Development. 2008;79(2):395–410. 10.1111/j.1467-8624.2007.01132.x 18366430

[29] Hoffman PT. Why Was It Europeans Who Conquered the World. The Journal of Economic History. 2012;72(03):601–33. 10.1017/S0022050712000319

[30] Lyons DE, Young AG, Keil FC. The Hidden Structure of Overimitation. Proceedings of the National Academy of Sciences of the United States of America. 2007;104(50):19751 10.1073/pnas.0704452104 18056814 PMC2148370

[31] Gruber T, Deschenaux A, Frick A, Clément F. Group Membership Influences More Social Identification Than Social Learning or Overimitation in Children. Child Development. 2017;0(0). 10.1111/cdev.12931 28846135

[32] Wood LA, Harrison RA, Lucas AJ, McGuigan N, Burdett ERR, Whiten A. “Model age-based” and “copy when uncertain” biases in children’s social learning of a novel task. Journal of Experimental Child Psychology. 2016;150:272–84. 10.1016/j.jecp.2016.06.005 27371768

[33] Wilks M, Collierbaker E, Nielsen M. Preschool children favor copying a successful individual over an unsuccessful group. Developmental Science. 2015;18(6):1014–24. 10.1111/desc.12274 25529854

[34] Dunham Y, Emory J. Of Affect and Ambiguity: The Emergence of Preference for Arbitrary Ingroups. Journal of Social Issues. 2014;70(1):81–98. 10.1111/josi.12048

[35] Howard LH, Henderson AM, Carrazza C, Woodward AL. Infants' and Young Children's Imitation of Linguistic In-Group and Out-Group Informants. Child Development. 2015;86(1):259 10.1111/cdev.12299 25263528 PMC4358791

[36] Whiten A, McGuigan N, Marshall-Pescini S, Hopper LM. Emulation, imitation, over-imitation and the scope of culture for child and chimpanzee. Philosophical Transactions of the Royal Society B: Biological Sciences. 2009;364(1528):2417–28. 10.1098/rstb.2009.0069 19620112 PMC2865074

[37] Frick A, Clément F, Gruber T. Evidence for a sex effect during overimitation: boys copy irrelevant modelled actions more than girls across cultures. Royal Society Open Science. 2017;4(12):170367 10.1098/rsos.170367 29308216 PMC5749984

[38] Legare CH, Nielsen M. Imitation and Innovation: The Dual Engines of Cultural Learning. Trends in Cognitive Sciences. 2015;19(11):688–99. 10.1016/j.tics.2015.08.005 26440121

[39] Chartrand TL, Lakin JL. The Antecedents and Consequences of Human Behavioral Mimicry. Annual Review of Psychology. 2013;64(1):285–308. 10.1146/annurev-psych-113011-143754 23020640

[40] Cordonier L, Nettles T, Rochat P. Strong and strategic conformity understanding by 3‐ and 5‐year‐old children. British Journal of Development Psychology. 2018;36(3):438–51. 10.1111/bjdp.12229 29265381

[41] Yu Y, Kushnir T. Social context effects in 2- and 4-year-olds’ selective versus faithful imitation. Developmental Psychology. 2014;50(3):922–33. 10.1037/a0034242 23978298

[42] Chudek M, Baron AS, Birch S. Unselective Overimitators: The Evolutionary Implications of Children's Indiscriminate Copying of Successful and Prestigious Models. Child Development. 2016;87(3):782–94. 10.1111/cdev.12529 27189405

[43] Buttelmann D, Bohm R. The Ontogeny of the Motivation That Underlies In-Group Bias. Psychological Science. 2014;25(4):921–7. 10.1177/0956797613516802 24474724

